# To handle the inflation of odds ratios in a retrospective study with a profile penalized log‐likelihood approach

**DOI:** 10.1002/jcla.23849

**Published:** 2021-05-27

**Authors:** I‐Shiang Tzeng

**Affiliations:** ^1^ Department of Research Taipei Tzu Chi Hospital Buddhist Tzu Chi Medical Foundation Taipei Taiwan; ^2^ Department of Statistics National Taipei University Taipei Taiwan

**Keywords:** clear cell renal cell carcinoma, methodology, monotone likelihood

Dear Editor,

We read with great interest the paper authored by Chen et al.^1^ entitled “Integrin α7 is overexpressed and correlates with higher pathological grade increased T stage advanced TNM stage as well as worse survival in clear cell renal cell carcinoma patients: A retrospective study,” published in January 2020 in the Journal of Clinical Laboratory Analysis. The authors aimed to explore the association of integrin α7 with clinicopathological characteristics and overall survival (OS) in patients with clear cell renal cell carcinoma (ccRCC) based on a retrospective study. They found that high tumor integrin α7 expression was an independent predictor of poor OS (*p *< 0.001) in a multivariate Cox proportional hazards regression model. Although the present study makes a valuable contribution to this area of research some methodological points need further clarification.

The authors compared the OS between tumor integrin α7 high expression and low expression groups and found that the crude hazard ratio (HR) of OS increased 4.453 times in patients with high integrin α7 expression levels compared to those with low integrin α7 expression levels (ccRCC patients with high expression [*n* = 69] vs. ccRCC patients with low integrin α7 expression [*n* = 110]; HR = 4.453; 95% confidence interval [CI]: 2.633–7.532). Furthermore another significant factor that affects the OS of patients with ccRCC is the node (N) stage. The univariate Cox regression results showed that the crude HR of OS increased 8.197 times in patients with N1 stage compared to those with N0 stage (ccRCC patients with N1 stage [*n* = 12] vs. ccRCC patients with N0 stage [*n* = 167]; HR = 8.197; 95% confidence interval (CI): 4.297–15.636). Several authors have argued that large effect estimates may result from special conditions in a data set and this is known as “monotone likelihood”.^2^, ^3^ Thus researchers cannot be certain about the true value of HR if the CI of the HR is too wide. The CI along with the large effect estimate suggests reconstruction of the interval estimation based on profile penalized log likelihood (PPL).^3^

To demonstrate this an analysis of a breast cancer data^4^ set with the coxphf package^5^ provided an implementation of the Firth's logistic regression. Of the 100 patients 74 were censored with a median follow‐up time of 78 months (range 11–172 months). The survival time of 100 patients and four potential risk factors were investigated. The four potential risk factors included tumor stage (T) nodal status (N) histological grading (G) and cathepsin D immunoreactivity (CD). We summarized the analysis results in Table 1 which shows that the HR and 95% CI of histological grading were shrinking after modification based on PPL. Figure 1 also shows the histological grading 95% CI which was estimated based on a PPL likelihood that was narrower than the 95% CI without modification.

**TABLE 1:** Summary of analysis of breast cancer data under Cox's proportional hazards regression model before and after modifying based on profile penalized log likelihood

Characteristic	HR	95% CI	*p*‐Value	Modified HR	95% CI	*p*‐Value
Tumor stage	3.593	1.343–9.616	0.0109*	3.402	1.363–9.472	0.0082*
Nodal status	2.576	1.120–5.926	0.0260*	2.507	1.120–5.833	0.0253*
Historical grading	100100000	0.000–Inf	0.9966	11.296	1.466–1451.946	0.0136*
Cathepsin D immunoreactivity	1.492	0.626–3.558	0.3670	1.488	0.627–3.512	0.3645

Abbreviations: CI, confidence interval; HR, hazard ratio; Inf, infinite.

* <0.05.

**FIGURE 1:**
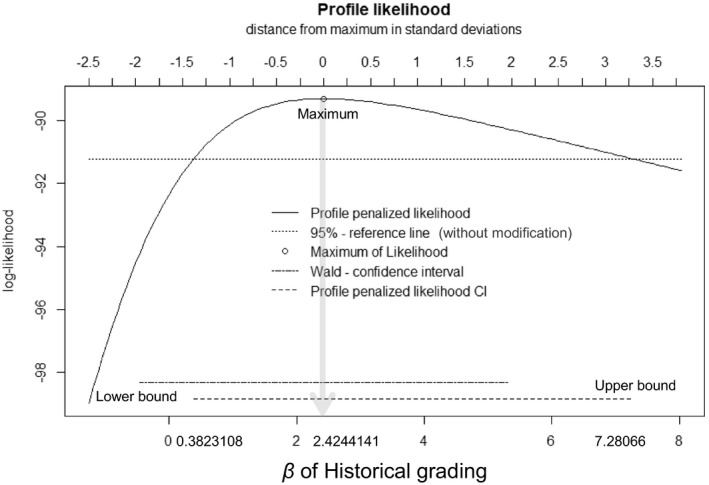
Plot of the profile penalized log‐likelihood function for variable historical grading

As shown in Table 1 in the study conducted by Chen et al. only 12 cases of patients with ccRCC were of N1 stage which means that their data were subject to sparse events and may increase the probability of the occurrence of monotone likelihood. We estimated that there will be fewer cases of patients with ccRCC with N1 stage due to patient survival (Chen et al. do not provide details). Different methods have been introduced to address the occurrence of monotone likelihood,but penalization through data augmentation is an efficient method that was published in 2001.^2^ After a multivariate analysis we found that the integrin α7 high expression factor (HR = 3.353; 95% CI: 1.900–5.918) preserved and the N1 stage factor did not in Cox's proportional hazards regression model (with forward stepwise method). We can conclude that the problem is not limited to univariate models if the N1 stage factor is preserved in the final multivariate model.^2^ The sample size number of events and magnitudes of association with the outcome degree of balance and number of binary covariates affect the prevalence of monotone likelihood when considering multivariate regression. The monotone likelihood phenomenon was observed in Cox's proportional hazards regression model. Hence we suggest that Chen et al. add to their text that a monotone likelihood limitation exists in their clinical data.

## DECLARATION OF FINANCIAL/OTHER RELATIONSHIPS

The authors of this letter have no relevant financial or other relationships to disclose.

## Data Availability

The data used to support the findings of this study are included within the article.
